# A Piezoelectric Sensor Based on MWCNT-Enhanced Polyvinyl Chloride Gel for Contact Perception of Grippers

**DOI:** 10.3390/biomimetics10060363

**Published:** 2025-06-03

**Authors:** Qiyun Zhong, Qingsong He, Diyi Liu, Xinyu Lu, Siyuan Liu, Yuze Ye, Yefu Wang

**Affiliations:** 1 Jiangsu Provincial Key Laboratory of Bionic Materials and Equipment, College of Mechanical and Electrical Engineering, Nanjing University of Aeronautics and Astronautics, Nanjing 210016, China; zhongqiyun@nuaa.edu.cn (Q.Z.); liudiyi2098@163.com (D.L.); lxy2405016@nuaa.edu.cn (X.L.); siyuanliu@nuaa.edu.cn (S.L.); yeyuze@nuaa.edu.cn (Y.Y.); 2 Key Laboratory of Advanced Technology for Small and Medium-Sized UAV, Ministry of Industry and Information Technology, Unmanned Aerial Vehicles Research Institute, Nanjing University of Aeronautics and Astronautics, Nanjing 210016, China; 3 State Key Laboratory of Mechanics and Control of Aerospace Structures, Nanjing University of Aeronautics and Astronautics, Nanjing 210016, China; 4 The First Affiliated Hospital of Nanjing Medical University, Nanjing 210029, China

**Keywords:** piezoelectric sensor, polyvinyl chloride gel, MWCNT-enhanced, KNN, contact perception

## Abstract

In contrast to traditional hydrogels, which are susceptible to water evaporation and structural degradation, non-hydrogel materials are engineered for superior stability and consistent performance. Here, we report an innovative piezoelectric polyvinyl chloride/multi-walled carbon nanotube polymer gel (PVC/MWCNT polymer gel, PMPG) with exceptional linearity (as low as 1.31%), high sensitivity (50–310.17 mV), rapid response (172–189 ms), and thermal stability. Under strain induction, ordered rearrangement of dipoles in PMPG and the enhancement of MWCNTs generate a potential difference. Increasing MWCNT content enhances output voltage, sensitivity, conductivity, maximum stress, Young’s modulus, and toughness, while reducing nonlinear error. Higher dibutyl adipate (DBA) content increases output voltage and slightly improves sensitivity but decreases mechanical strength. The optimal PMPG (PVC:DBA = 1:5, 1 wt% MWCNTs) exhibited outstanding performance. It exhibits a nonlinear error as low as 1.31%, a conductivity of 25.4 μS/cm, an 80% compressive strain tolerance (273 kPa stress), and dimensional stability for 90 days in air. By integrating PMPG with machine learning algorithms, soft robotic grippers gain advanced contact perception capabilities, enabling applications in medicine, rescue, exploration, and other fields requiring fine manipulation and adaptability. This work highlights PMPG’s potential as a stable, high-performance material for soft robotics and beyond.

## 1. Introduction

The field of flexible sensors has experienced exponential growth, emerging as a pivotal segment within the intelligent industry. Their lightweight, high degree of integration, and broad applicability have been principal impetuses of this rapid development. Flexible sensors have firmly established themselves in a wide and intricate range of applications, playing an integral part in various fields such as human motion and health monitoring [1,2], robotics [3,4], human–computer interaction [5,6], and biomedicine [7,8]. Up to now, various flexible sensors have been proposed to detect mechanical signals, including piezoresistive [9,10], capacitive [11,12], piezoelectric [13,14], and triboelectric [15]. Among various types of flexible sensors, flexible piezoelectric sensors stand out for their unique piezoelectric effect, which can directly convert mechanical energy into electrical energy, featuring self-powering and fast response capabilities [16]. For example, in the field of energy harvesting, the self-powering property of flexible piezoelectric sensors enables them to convert ambient mechanical vibrations into usable electrical energy [17,18]. In biomechanical monitoring, their fast response to small pressure and strain enables them to detect human movements and physiological signals [19,20,21]. Previous research has explored a variety of materials for flexible piezoelectric sensors, such as polymers like polyvinylidene fluoride (PVDF) and its copolymers, which offer good flexibility but often suffer from limited mechanical strength [22,23]. Some studies have incorporated metal nanoparticles or conductive polymers to enhance conductivity, yet these additives may compromise the flexibility and long-term stability of the sensors [24,25,26,27]. Additionally, while geometric and multilayer design strategies have optimized sensor structures [28,29,30,31], and innovative manufacturing processes have improved performance and reliability [32,33], there remains a pressing need for materials that can simultaneously enhance linearity, response speed, flexibility, and stability. Carbon-based materials, especially multi-walled carbon nanotubes (MWCNTs), have attracted significant attention due to their unique properties. MWCNTs possess outstanding electrical conductivity, high aspect ratio, and exceptional mechanical strength, which can potentially address the limitations of existing materials [34,35,36]. Compared with other commonly used conductive fillers, such as graphene or carbon black, MWCNTs can form more efficient conductive networks with fewer additives, reducing the impact on the matrix’s flexibility. Moreover, their one-dimensional structure allows for better dispersion within the polymer matrix, improving the uniformity of the composite material and sensor performance.

The objective of this study is to develop a novel flexible piezoelectric sensor with excellent mechanical and electrical properties. In this study, an innovative material polyvinyl chloride/multi-walled carbon nanotube polymer gel (PVC/MWCNT polymer gel, PMPG) is introduced. This composite boasts remarkable attributes, including superb thermal stability, excellent linearity, good sensitivity, swift response, and enduring durability. The PMPG is meticulously crafted through a process of physical crosslinking, utilizing PVC gel as the elastomeric matrix and multi-walled carbon nanotubes (MWCNTs) as the dielectric component. By modulating the concentrations of dibutyl adipate (DBA) and MWCNTs, mechanical and electrical properties of PMPG can be adjusted to suit specific applications. To substantiate the practicality of PMPG sensor, it has been integrated into a robotic gripper. This integration allows for dynamic interaction with a variety of objects through a repertoire of grasping and releasing maneuvers. Employing a K-nearest neighbors (KNN) machine learning algorithm, the gripper’s sensory feedback is adeptly learned and categorized. The incorporation of haptic feedback into the soft gripper not only augments its intelligence and autonomy but also significantly extends its utility in unstructured environments.

## 2. Materials and Methods

### 2.1. Material

Polyvinyl chloride (PVC) was purchased from Aladdin Biochemical Technology Co., Ltd. (Shanghai, China). PVC has a purity of 99.9%, with a weighted-average molecular weight (Mw) of 233,000 and a number-average molecular weight (Mn) of 99,000. The plasticizer dibutyl adipate (DBA, 99% purity) was purchased from Shanghai McLean Biochemical Technology Co., Ltd. (Shanghai, China). The organic solvent tetrahydrofuran (THF) was purchased from Shanghai Aladdin Biochemical Technology Co., Ltd. (Shanghai, China). Multi-walled carbon nanotubes (>95% purity, inner diameter 3–5 nm, outer diameter 8–15 nm, length 3–12 μm) were purchased from Suzhou Carbonfund Graphene Technology Co., Ltd. (Suzhou, China). The plasticizer of the encapsulation material, diisononyl cyclohexane 1,2-dicarboxylate (Hexamoll DINCH, 99% purity)), was provided by Nanjing Chemical Reagent Co., Ltd. (Nanjing, China). All materials were used directly after reception without any purification.

### 2.2. Preparation of PMPG

Dibutyl adipate (DBA) and tetrahydrofuran (THF) were blended in a ratio of 3:5 and agitated in a magnetic stirrer at 400 r/min for 10 min. Subsequently, polyvinyl chloride (PVC) pellets with a PVC:DBA ratio of 1:5 were gradually introduced into the mixed solution and subjected to ultrasonic treatment for 10 min. The treated mixture was then stirred using a magnetic stirrer at 1500 r/min until the PVC particles were entirely dissolved. Next, 1 wt% of multi-walled carbon nanotubes (MWCNTs) relative to the total mass of PVC and DBA was incorporated into the mixture after increasing the rotational speed to 400 r/min. Thereafter, the rotational speed was readjusted back to 1500 r/min, and following 48 h of stirring, the obtained mixed solution was poured into a Petri dish and placed in a fume hood for curing over a period of 36 h. Eventually, PMPG with a PVC:DBA ratio of 1:5 and an MWCNT content of 1 wt% was obtained. In this paper, PMPG samples with four distinct plasticizer ratios (PVC:DBA = 1:3, PVC:DBA = 1:5, PVC:DBA = 1:7, PVC:DBA = 1:9) and four different carbon nanotube contents (0.5 wt%, 1 wt%, 1.5 wt%, 2 wt%) were prepared and investigated (Table S1).

### 2.3. Characterization and Analysis

The FTIR test was conducted using a FTIR spectrometer (Nicolet iS50R, Thermo Fisher Scientific, Waltham, MA, USA). The test was carried out within a wavelength range of 400 cm⁻^1^ to 4000 cm⁻^1^, with a step size of 4 cm⁻^1^ and a sample scan number of 32. For the TG-DSC test, a synchronous thermal analyzer (NETZSCH STA 449 F3 Jupiter, NETZSCH, Selb, Germany) was employed. The temperature range was set from 30 °C to 800 °C, and the heating rate was 20 °C/min under a nitrogen atmosphere. The electron microscopy imaging was conducted using a ZEISS GeminiSEM 300 (Oberkochen, Germany). The XRD test was conducted using an X-ray diffractometer (Rigaku SmartLab SE, Tokyo, Japan). The test was performed with a scanning angle range of 10–60° and a scanning speed of 2°/min. For electrochemical testing, an electrochemical workstation (CHI604E, Shanghai Chenhua Instruments, Shanghai, China) was utilized. The mechanical performance test used an electronic universal testing machine (HZ-1007E, Dongguan Li Xian Instrument Technology Co., Ltd., Dongguan, China). The electrical test platform consists of PMPG, an NI USB-6001 data acquisition card (Shanghai Enable Instruments Co., Ltd., Shanghai, China), and the host computer. The two ends of PMPG were laminated with copper foil electrodes and connected to the analog input of the data acquisition card, and the voltage signal was collected by a single-ended connection. The data acquisition card communicates with the computer through the USB interface, and LabVIEW is used for host computer development and data processing.

## 3. Results and Discussion

### 3.1. Design Preparation and Characterization Analysis

**Figure 1:**
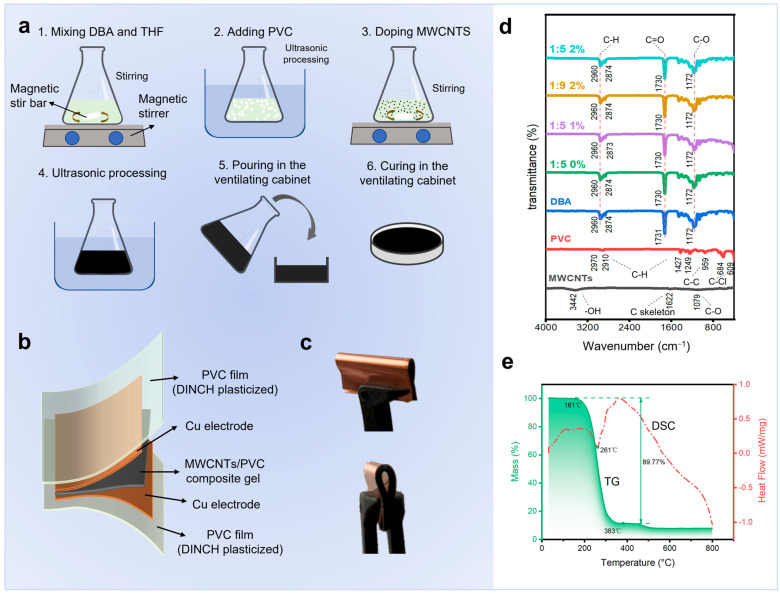
Preparation and characterization of PMPG and sensor demonstration. (**a**) Preparation process of PMPG. (**b**) Schematic of sensor constructional decomposition and layering and demonstration of bending performance. (**c**) Bending of PMPG flexible sensors. (**d**) Fourier infrared spectra of PMPG and each component (MWCNTs, PVC, DBA, 1: 5 0 wt%, 1: 5 1 wt%, 1: 9 1 wt%, 1: 5 2 wt%). (**e**) Simultaneous thermal analysis of PMPG.

The fabrication process of PMPG is shown in Figure 1a. The specific steps are shown in the experimental section. The flexible piezoelectric sensor based on PMPG shown in Figure 1b adopts a “sandwich” structure in which a flexible Cu electrode is placed on each side of a piece of PMPG, and a poly(vinyl chloride) (disononyl cyclohexane-1,2-dicarboxylate, DINCH plasticized) film is added outside of the electrode to serve as an insulating layer for the encapsulation of the piezoelectric sensor. As shown in Figure 1c, the encapsulated PMPG sensors are flexible enough to be bent and twisted arbitrarily to a radius of less than 1 mm.

The FTIR results of PMPG and its components are shown in Figure 1d. In the FTIR spectrum of MWCNTs, the peak at 1622 cm^−1^ is caused by the vibration of the carbon skeleton. The peak at about 1079 cm^−1^ is generated by the C-O stretching vibration and the peak at 3442 cm^−1^ is generated by the bending vibration of -OH, which occur due to the presence of certain impurities in MWCNTs and the existence of little oxidized multi-walled carbon nanotubes. In the FTIR spectra of PVC, the C-H asymmetric stretching vibration peaks on the formate polymer chain appeared at 2910~2970 cm^−1^, and the C-H symmetric stretching vibration peaks appeared at 1249~1427 cm^−1^. The absorption peak at 959 cm^−1^ is caused by the vibration characteristic of the C-C skeleton of PVC, and the peaks in the range of 609 cm^−1^ to 684 cm^−1^ are caused by the C-Cl stretching vibration. The FTIR spectrum of DBA shows three distinct absorption bands, with C-H symmetric and asymmetric stretching vibration peaks in the range of 2874~2960 cm^−1^ for methyl and methylene, as well as C=O and C-O stretching vibration peaks near 1731 cm^−1^ and 1172 cm^−1^, respectively. The FTIR spectra of PMPGs with different composition ratios (PVC:DBA = 1:5, 0 wt% of MWCNTs; PVC:DBA = 1:5, 1 wt% of MWCNTs; PVC:DBA = 1:9, 1 wt% of MWCNTs; PVC:DBA = 1:5, 2 wt% of MWCNTs) contained all the characteristic peaks or superimposed peaks associated with PVC and DBA and showed no new absorption peaks. In PMPG, the characteristic peaks of C skeleton (1622 cm^−1^) and C-O (1079 cm^−1^) of MWCNTs overlap with the peaks of C=O (1731 cm^−1^) and C-O (1172 cm^−1^) of DBA to a certain extent. The characteristic peaks of PMPG contain the characteristic peaks of PVC, DBA, and MWCNTs and no new characteristic peaks were generated. These indicate that there is no substantial chemical bonding between PVC, DBA, and MWCNTs, suggesting that the PVC polymer chains are filled and surrounded by DBA and MWCNTs in a physically mixed state. FTIR spectroscopy fails to fully characterize the material’s microstructure. Future studies should incorporate multiple techniques, including Raman spectroscopy, to systematically analyze the material composition, thereby enhancing the accuracy and reliability of the results.

The TG curves (Figure 1e and Figure S1) and thermal analysis results (Table 1) provide a comprehensive understanding of the thermal behavior of PMPG and its components. For pure PVC, it exhibits two distinct pyrolysis zones. The first zone ranges from 229 to 407 °C, and the second zone spans from 407 to 563 °C. DBA, on the other hand, has a pyrolysis zone spanning 90~281 °C. For PMPG (PVC:DBA = 1:5, 1 wt% MWCNTs), its pyrolysis zone is 161~383 °C. The TG curve of PMPG shows a mass loss rate of 89.77%. Therefore, PMPG does not decompose and exhibits thermal stability below 161 °C. From the DSC curve, an obvious heat absorption peak emerges at approximately 261 °C. This peak indicates that the melting temperature of the gel–elastomer network in PMPG lies between 161 °C and 261 °C (Figure 1e), marking the phase transition process from a highly elastic state to a viscous–flow state. Subsequently, the melt–decomposition process dominates, mainly involving the dechlorination of PVC. In summary, the TG-DSC curves of PMPG demonstrate no phase transition in the range of 30 °C~161 °C. This absence of phase change within this critical temperature interval not only showcases its good thermal stability but also highlights its superiority as a flexible sensing material.

The study on the recyclability of PMPG (polyvinyl chloride/multi-walled carbon nanotube polymer gel) is of great significance. In traditional methods, pyrolysis decomposes PVC into monomers or oligomers through anaerobic heating, enabling the recycling of PVC and the reuse of MWCNTs. Solvent extraction utilizes solvents such as tetrahydrofuran to separate and recycle the materials based on the difference in solubility between PVC and MWCNTs [37]. In recent studies, new ideas have been proposed [38,39], such as using ionic liquids to promote the dechlorination of PVC or borrowing the strategy of co-upcycling of mixed plastics to improve the recycling efficiency. However, currently, there are still challenges such as difficulties in material separation, high environmental risks, and high recycling costs. In the future, it is necessary to develop new separation technologies, explore environmentally friendly processes, reduce costs, and optimize performance to promote the large-scale application of PMPG in the sustainable field.

**Figure 2:**
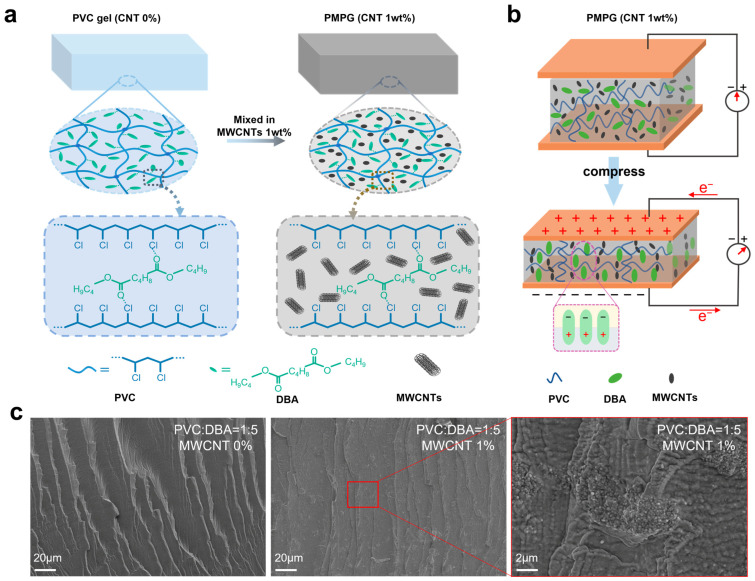
Structure and sensing mechanism of PMPG. (**a**) Structure of PMPG. (**b**) Sensing mechanism of PMPG. (**c**) SEM images of PVC gel and PMPG.

XRD analysis of PMPG (Figure S2) indicates an amorphous structure, primarily characterized by broadened diffraction peaks from PVC (2θ ≈ 20°) [40,41], while the crystalline signals of MWCNTs (2θ ≈ 26.5°) [42,43] are not significantly detected, likely due to their low content (1 wt%) and dispersion in the matrix. The plasticization effect of DBA reduces the crystallinity of PVC, enhancing the flexibility of molecular chains and facilitating mechanical deformation, which is beneficial for strain-induced dipole alignment (Figure 2b). In terms of mechanical properties, the amorphous matrix and conductive networks of MWCNTs synergistically improve the flexibility and strength of the material. Electrochemically, low crystallinity facilitates dipole polarization, while MWCNTs accelerate charge migration, endowing the material with high linearity. Structural analysis provides a theoretical basis for the sensing mechanism, but the quantitative relationship between crystallinity and modulus requires further verification by DMA.

### 3.2. Sensing Mechanism Analysis

The PMPG is filled with PVC, MWCNTs, and DBA molecules, as shown in Figure 2a. PVC is a polar polymer material due to the presence of chlorine atoms in its molecular structure. The chlorine atoms in the PVC molecule form polar covalent bonds with carbon atoms, and the polarity comes from the difference in electronegativity between chlorine atoms and carbon atoms [44]. In DBA molecules, the carbon–oxygen double bond (C=O) and single bond (C-O) in the ester group cause uneven charge distribution, polarizing the molecule. Additionally, the asymmetric structure of DBA renders the entire molecule polar [45]. The ester groups (-COO-) of DBA interact with the chlorine atoms (-Cl) of PVC via dipole–dipole interactions in PMPG.

In the absence of an external force, the overall dipole within PMPG is randomly oriented, resulting in a net electric dipole moment of zero. Upon external mechanical stimulation, the distance between the PVC and DBA molecular chains decreases, inducing a more ordered arrangement of the molecular chains, as shown in Figure 2b. This force-driven rearrangement triggers spontaneous polarization, causing electric dipoles to orient in a more ordered manner and generating a potential difference between the upper and lower electrodes [46]. As the applied pressure increases, the dipole moments become increasingly ordered, amplifying the potential difference.

However, pure PVC gel exhibits a negligible potential difference under pressure, necessitating the addition of MWCNTs for improvement. MWCNTs form conductive networks in PMPG, enhancing its electrical conductivity and enabling faster charge migration [47]. As shown in Figure 2c, the SEM image of the PVC gel (PVC:DBA = 1:5) shows that its surface is relatively smooth, with no obvious bumps or irregular structures. The surface of the PMPG (PVC:DBA = 1:5, 1 wt% of MWCNTs) shows some bumps and irregular structures. The latter images further show the details of these irregular structures, and it can be seen that there are some fine, fiber-like or granular materials distributed on the surface, which are MWCNTs and their agglomerates.

The EDX spectrum (Figure S3 and Table S2) exhibits dominant peaks for carbon (74.21 wt%), chlorine (23.05 wt%), and oxygen (2.74 wt%), consistent with the compositions of PVC, DBA, and MWCNTs. The carbon content in EDS significantly exceeds the theoretical sum of pure PVC (approximately 38.4 wt% C) and DBA (approximately 65.1 wt% C), verifying the presence of MWCNTs in the protrusions and granules observed in the images. However, due to their low content (1 wt%), MWCNTs do not form independent peaks in the EDS spectrum but are superimposed on the matrix carbon signal.

### 3.3. Electrical and Electrochemical Characterization

**Figure 3:**
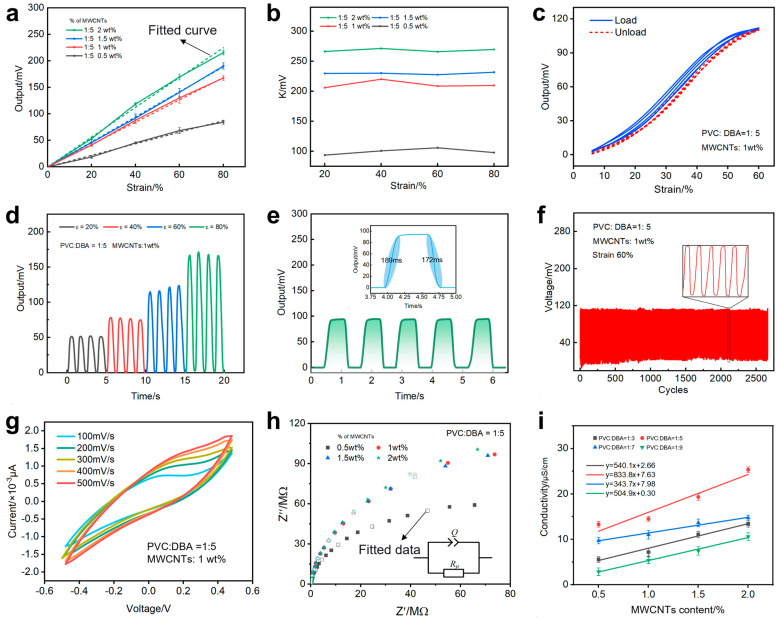
Electrical and electrochemical characterization of PMPG. (**a**) Linearity of PMPG with different MWCNT contents. (**b**) Sensitivity of PMPG with different MWCNT contents. (**c**) Hysteresis properties of PMPG. (**d**) Compressive properties of PMPG with different strains. (**e**) Response time of PMPG. (**f**) Stability of 2500 cycles of stretching. (**g**) Cyclic voltammetry, (**h**) electrochemical resistance Nyquist plots, and (**i**) electrical conductivity of PMPG.

Linearity, a critical metric for sensor devices, measures the deviation of the output-input characteristic curve from a fitted straight line under stable input conditions [48]. It is quantified by the nonlinear error, with a lower nonlinear error indicating superior linearity in a PMPG sensor. The nonlinear error represents the maximum deviation of the sensor’s output–input curve from the straight line, expressed as a percentage of the full-scale output, and is defined b Equation (1):

γ_L_ = ±ΔL_max_/Y_FS_ × 100%, (1)

where γ_L_ is the nonlinear error, ΔL_max_ is the maximum relative error, and Y_FS_ is the full-scale output. With a constant ratio of PVC to DBA, the output voltage of PMPG rises with the increase of compressive strain because the conductive network inside the material undergoes reconstruction during deformation, resulting in a more compact and efficient conductive pathway (Figure 3a, Figures S4 and S5) [49,50]. The output voltage increases with the increase in the mass fraction of MWCNTs, which is due to the increase in the content of MWCNTs that makes the conductive network inside the material more continuous and stable. The output voltage also increased with the increase in DBA content, which was attributed to the fact that DBA, as a plasticizer, improved the flexibility and deformation ability of the material and promoted the reconstruction of the conductive network (Figure 3a, Figures S4 and S5). The nonlinear error of PMPG diminishes with the increment in MWCNT content because the conductive network inside PMPG becomes more continuous and homogeneous with the increase of MWCNTs concentration. Optimal linearity and the lowest nonlinear error of 1.31% were achieved when the PVC:DBA ratio was 1:9 and the MWCNT content was 2 wt%. When the ratio of PVC to DBA was 1:5, the PMPG’s demonstrated low nonlinear errors (1.76–3.80%) at different MWCNT contents (Table S3). Sensitivity [51] escalates with the increase in MWCNT content and DBA content (Figure 3b and Figure S6). The gel’s peak sensitivity reached a maximum of 310.17 mV at an MWCNT concentration of 2 wt% and a PVC:DBA ratio of 1:9 (Figure 3b and Figure S6), which reflects that the introduction of MWCNTs positively contributes to the sensitivity enhancement of the PMPG. These observations may be attributed to conducting networks caused by MWCNTs [52]. Notably, the sensitivity of the PMPG containing 1 wt% MWCNTs was more than double that of the gel with 0.5 wt% MWCNTs, which indicates that the sensitivity increases significantly when the MWCNT content reaches 1 wt% and above. The sensitivity of PMPG (Equation (S1)) remained relatively constant with the rise in strain level at a PVC:DBA ratio of 1:5, yet it displayed a marked upward trend with the increase in MWCNT content. Figure 3c depicts the output voltage versus strain curves for the PMPG (PVC:DBA = 1:5, 1 wt% of MWCNTs) during the pressing and unloading phases over five cycles of compression. The slight divergence in these curves suggests the presence of a hysteresis effect. The output voltage during the loading phase is greater than that of the unloading phase, potentially due to the elastomeric nature of the PMPG and its inherent mechanical attributes that lead to the difference in the electrical response. The hysteresis error (Equation (S2)), shown in Figure S7 and Table S4, initially decreases then increases with the rise in DBA content at a constant 1 wt% MWCNTs and 60% strain. The hysteresis error decreases with higher MWCNT content under fixed DBA concentration and strain levels, whereas it increases with strain at constant MWCNT and DBA concentrations [53,54]. The specimen (h) with a PVC:DBA ratio of 1:5, 2 wt% MWCNTs, and 60% strain exhibits the lowest hysteresis error of just 5.51%, indicating that the high MWCNT content and moderate DBA ratio can reduce the hysteresis errors. The influence of varying strains on the stability of the PMPG output is further examined in Figure 3d. A slight fluctuation in the output voltage signal is observed with increasing strain, possibly attributed to the defects within the polymer network and the charge migration’s susceptibility to the hysteresis effect. Given its stable sensitivity (about 230 mV), excellent linearity (3.32%), and low hysteresis error (5.51%), the PMPG formulation with a PVC:DBA ratio of 1:5 and 1 wt% MWCNTs was selected for response time and fatigue stability tests, as shown in Figure 3e,f. The PMPG exhibits a rapid response time of only 189 ms and demonstrates commendable stability and reliability over 2500 cycles. Additionally, the response characteristics of the PMPG at different compression speeds were scrutinized. An increase in speed leads to a certain degree of output signal fluctuation (Figure S8), potentially arising from the PMPG’s mechanical properties. Moreover, the sensing gel’s deformation may not promptly recover during rapid loading and unloading, contributing to these fluctuations.

To evaluate the electrochemical property and conductivity of the PMPG, a cyclic voltammetry test was conducted, as depicted in Figure 3g. The absence of distinct oxidation and reduction peaks within a single charge/discharge cycle spanning −0.5 V to 0.5 V at various scan rates suggests that the MWCNT and DBA within the gel exhibits commendable stability and does not react chemically under voltage. Further exploration of the gel’s electrochemical impedance was undertaken, with results presented in the Nyquist plot (Figure 3h) and the Bode plot (Figure S9). The Nyquist plot’s capacitive impedance arcs for the sensing material’s electrode system in the first quadrant are circular arcs, aligning with the impedance frequency response of a composite element (RQ). The constant phase angle element’s characteristics reveal that Q represents resistance when n equals 0 and pure capacitive behavior when n equals 1. As the index n increases, the capacitive nature of element Q intensifies. The exponent n of the constant phase angle element Q is positively correlated with the MWCNT content, indicating that the charge migrates between the electrodes of the PMPG (Tables S5–S8). Higher MWCNT content leads to greater charge accumulation, enhancing the capacitive characteristics of element Q. The Bode plot illustrates that below 10^4^ Hz, the electrode system’s phase angle increases, with a drop in impedance and capacitance. Within a perturbation frequency range of 10^4^ Hz~10^6^ Hz, the phase angle of the electrode system is close to 90°, and the impedance mode nears zero, indicating a decrease in system resistance. At this frequency range, the impedance is predominantly capacitive reactance, with high polarization resistance Rp (Tables S5–S8). The conductivity of PMPG is calculated using Equation (S3) from the Supplementary Material, with data fitting results displayed in Figure 3i and Table S9. The conductivity of the PMPG increased with the increase in MWCNTs. The incorporation of MWCNTs augments PMPG’s conductivity. The conductivity of the PMPG increases and then decreases with the increase in DBA content. When PVC:DBA = 1:5, the conductivity of PMPG is higher than the other ratios, which may be due to the fact that a proper DBA content will facilitate the formation of conductive pathways. And the density of MWCNTs in the network of PVC polymer decreases when the content of DBA is high, which in turn weakens the conductivity of PMPG. By linear fitting, the conductivity showed a clear linear relationship with the content of MWCNTs. At a 2 wt% MWCNT content, PMPG’s conductivity ranges from 10.6 μS/cm to 25.4 μS/cm, marking a more than thirtyfold increase compared to PVC gels devoid of MWCNTs, which exhibit conductivities between 0.31 μS/cm and 0.36 μS/cm [20].

### 3.4. Mechanical Characterization

**Figure 4:**
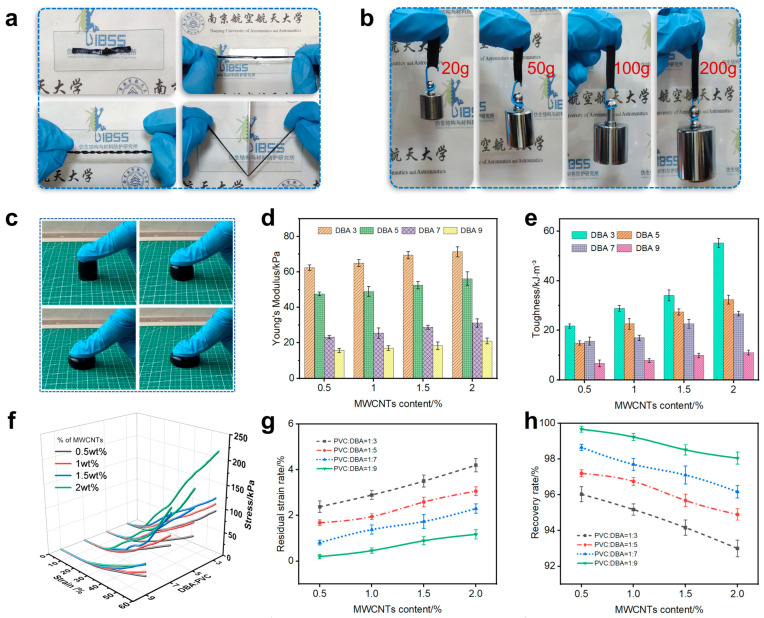
Mechanical characterization of PMPG. (**a**) Knotting, torsion, puncture, and shear resistance tests; (**b**) lifting load test; (**c**) compression performance; (**d**) Young’s modulus; (**e**) toughness; and (**f**) stress–strain graph of PMPG. (**g**) Residual strain ratio and (**h**) recovery rate after compression.

PMPG exhibits exceptional flexibility and tunable mechanical properties. To underscore its pliability, we demonstrate a range of macroscopic deformations. As illustrated in Figure 4a, PMPG can be deformed and stretched into various shapes, yet it retains the ability to expand and return to its original form. It also demonstrates resilience against sharp objects, showcasing superior puncture and shear resistance. Various weights were applied to the PMPG, which elongated progressively without breaking, indicative of its robust tensile strength (Figure 4b). Furthermore, PMPG maintains impressive resilience even after compression (Figure 4c). The Young’s modulus (Table S10), toughness (Table S11) and maximum stress of PMPG increase with the rise in MWCNT content, as shown in Figure 4d–f, respectively. When MWCNTs are dispersed in PMPG, they effectively transfer and disperse external stresses, thereby improving the mechanical properties of the material. Conversely, these properties decrease with an increase in DBA content. This suggests that MWCNTs significantly enhance the mechanical properties of the material, while DBA has a diminishing effect. This reduction is attributed to an excessive plasticizer occupying the gel’s solid space, leading to a sparser polymer network and reduced mechanical properties. Unlike metallic materials, PMPG’s stress–strain curve lacks a distinct yield phase, with stress increasing as strain increases. When PVC: DBA = 1:3 and the MWCNT content is 2 wt%, PMPG exhibits a maximum stress of 273 kPa at 80% compressive strain. Under identical proportioning conditions, PMPG’s Young’s modulus (linear elasticity phase) reaches 71.3 kPa, significantly higher than other variants, indicating that higher DBA content softens PMPG, with the opposite effect observed for MWCNTs. And the PMPG with this ratio exhibits a toughness value of 55.29 kJ/m^3^, demonstrating excellent resistance to tensile and shear stresses. The high weighted-average molecular weight (Mw = 233,000) and broad molecular weight distribution (Mw/Mn ≈ 2.35) of poly(vinyl chloride) (PVC) synergistically enhance the elastomeric properties of the PMPG. Extensive chain entanglements formed by high-Mw chains provide abundant physical crosslinking points, promoting elastic recovery. The coexistence of short and long chains in the broad distribution achieves a balance between segmental mobility and network integrity, endowing the material with both flexibility and mechanical robustness. As shown in Figure 4g,h, the residual strain ratio (the ratio of non-recoverable deformation to initial height) increases with MWCNT content yet decreases with DBA concentration, whereas the recovery rate (the ratio of recovery to the amount of compression) shows the inverse relationship. The results indicate that MWCNTs suppress the material’s elastic recovery, whereas DBA improves its flexibility. At the lowest MWCNT content (0.5 wt%), PMPG with PVC:DBA = 1:9 offers the best rebound effect, with a minimal residual strain ratio of 1.2% (Figure 4g) and a recovery rate of up to 99.7% (Figure 4h). In contrast, PMPG with PVC:DBA = 1:3 results in a higher residual strain ratio of 4.2% (2 wt% of MWCNTs) and a slightly lower recovery rate of 96.0% (0.5 wt% of MWCNTs). It is evident that the decrease in MWCNT content and the increase in DBA content reduce the mechanical properties of the sensing material, but they also impart greater softness and elasticity, aiding in the material’s shape recovery post deformation.

### 3.5. Detections of Robotic Grasping Using Auxetic PMPG Sensors

**Figure 5:**
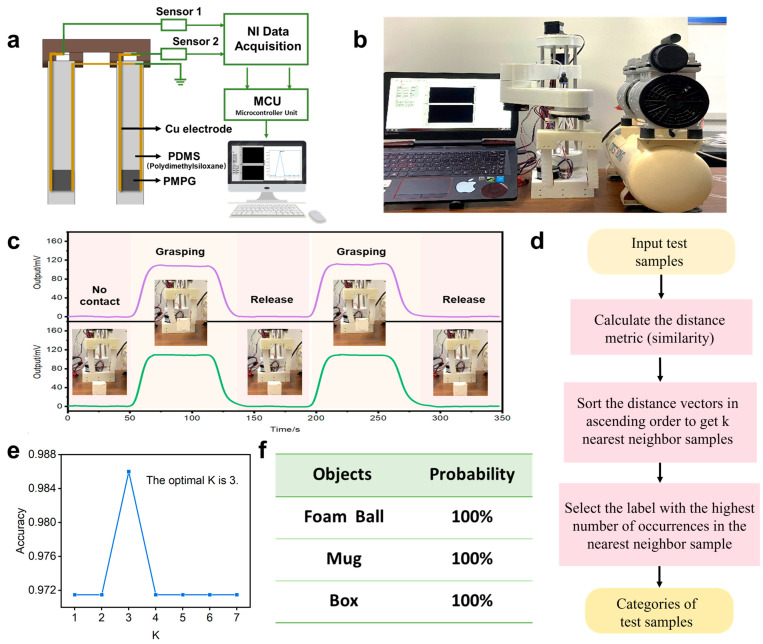
Structure and application of PMPG-based tactile sensing array system integrated on soft gripper. (**a**) Schematic diagram of the tactile sensing system. (**b**) Physical image of the tactile gripper and the signal monitoring system diagram. (**c**) Real-time voltage signal response of the sensor to monitor the grasping motion of the gripper. (**d**) The structure construction of our KNN mole. (**e**) The accuracy corresponding to different K values. (**f**) Actual gripping prediction results.

To underscore the superior performance of the PMPG sensor and the efficacy of the detection methodology, the sensing element has been integrated into a robotic gripper. This gripper engages with a variety of objects through a series of grasping and releasing actions. The grasping tasks are preprogrammed using a bionic two-fingered flexible manipulator, with the grasping state being analyzed through strain data captured by the sensor. As shown in Figure 5a, the sensors (PVC:DBA = 1:5, 1 wt% MWCNTs), which demonstrate stable sensitivity, excellent linearity, rapid response, and low hysteresis error, are affixed to the tips of each bionic finger to monitor the grasping state. Building upon this, we have assembled a real-time signal reception system that displays the output signals of the haptic sensing elements, as depicted in Figure 5b. The system comprises an acquisition unit and a host computer. The acquisition unit is tasked with signal acquisition and filtering, while the host computer analyzes the sensing signals to achieve real-time data visualization. As illustrated in Figure 5c, a sharp increase in output voltage is observed upon the gripper’s contact with an object, attributable to the increased force exerted by the gripper, enhancing the coupling between the sensor and the object. During the gripping phase, the output remains stable, and upon releasing the object, the output reverts to its initial state. This application exemplifies the sensor system’s capability to interact with objects, highlighting its potential for sensing and interaction in diverse environments.

The flexible tactile sensing element, integrated onto the soft gripper, is capable of sensing and providing feedback on pressure during object manipulation. However, given the nonlinearity of the sensing element, machine learning algorithms are employed to learn and classify the feedback information from different target objects, monitor the grasping state, and facilitate the recognition of captured objects. We have adopted the K-nearest neighbor (KNN) algorithm, and Figure 5d illustrates the structure of our KNN model. Initially, the sample data to be tested is inputted, and the distance between the samples is calculated. The distance vector is then sorted in ascending order, identifying the first K samples with the smallest distance vector and highest similarity as the K-nearest neighbor samples of the test sample, forming a cluster. Subsequently, the frequency of category selection within each cluster is tallied, with the most frequently selected category determined as the category of the corresponding test sample. In this context, similarity is gauged by the Mahalanobis distance, a measure of the distance between a point and a distribution, suitable for assessing the similarity of two unknown sample sets. The Mahalanobis distance *D_M_*(*x*) for a single data point can be expressed as a multivariate vector with mean μ and covariance matrix Σ, i.e.,

M(x) = [(x − μ)′Σ−1(x − μ)]½,(2)

where Σ is the covariance matrix of the multidimensional random variable and μ is the mean of the sample. According to Equation (2), when the covariance matrix is the identity matrix, the Mahalanobis distance equates to the Euclidean distance. Additionally, the K value is pivotal for determining the weight of neighboring samples and ensuring the accuracy of category prediction. A small K value results in fewer samples for model training, potentially leading to low classification accuracy, whereas a large K value, with too many samples, may result in underfitting. Therefore, we select the optimal K value through cross-validation, as shown in Figure 5e. The results indicate that the best K value is 3, yielding the highest prediction accuracy of 98.6%. A model is then constructed based on this value to predict the classification of the test samples, and the model’s prediction accuracy is evaluated. To validate the feasibility of the grasping strategy based on the KNN algorithm, grasping training and testing are conducted on an experimental platform. The experimental environment for feedback recognition in this section utilizes PyCharm on Windows 10, with Python 3.9 as the programming language, and the intermediate data acquisition and processing tasks are managed by an upper computer interface developed in LabVIEW. The target objects for this experiment include disposable water cups, foam balls, and headphone boxes. Post execution of the KNN algorithm, the prediction results, as shown in Figure 5f, confirm a model evaluation accuracy of 100%, with the program’s predictions perfectly aligning with actual outcomes. This outcome further substantiates the KNN algorithm’s viability for object grasping recognition experiments and offers insights into the integration of machine learning with flexible sensors.

Compared with similar materials reported in the literature (Table 2), the PMPG developed in this study shows advantages in durability, response time, conductivity and mechanical properties. Specifically, this material exhibits excellent cycling stability, capable of withstanding 2500 compression cycles, surpassing the performance of similar materials [55,56,57,58]. Its measured response time is 189 milliseconds, outperforming SiO₂@PANI (300 milliseconds) and PAM-oxCNTs (300 ms) [56,58]. Additionally, at 80% strain, the compressive stress of PMPG is 273 kPa, demonstrating superior mechanical properties to PHEMA/PPy gel [57], with well-balanced mechanical characteristics. The conductivity is at a moderate level, lower than that of P(AM/LMA) and PAM-oxCNTs [56,58], similar to PPy/CNT [59], and higher than the reference material PDA [60]. Future research could focus on enhancing conductivity and response speed through composite crosslinking strategies and structural optimization to expand its potential applications in flexible electronics and wearable devices.

## 4. Conclusions

In this study, a flexible piezoelectric sensing material (PMPG) with high linearity, sensitivity, rapid response, and thermal stability was successfully prepared via a simple casting method. Upon external mechanical stimulation, ordered rearrangement of dipoles in PMPG, together with MWCNTs enhancing the response, generates a potential difference between electrodes. The sensitivity, conductivity, and response time of PMPG span a range from 50 to 310.17 mV, 2.89 to 25.4 μS/cm, and 172 to 189 ms, respectively, with the absolute minimum nonlinear error being as low as 1.31%. By tuning MWCNT and DBA contents, precise regulation of electrical–mechanical properties was achieved. As the MWCNT content escalates, there is a corresponding increase in the output voltage, sensitivity, electrical conductivity, maximum stress, Young’s modulus, and toughness of PMPG, while the nonlinear error diminishes. Furthermore, with an increase in the content of DBA, the output voltage, sensitivity, and recovery rate of PMPG increase, but its maximum stress, Young’s modulus, toughness, and residual strain ratio decline. The electrical conductivity of PMPG initially increases with DBA content but then shows a decrease. The optimal PMPG (PVC:DBA = 1:5, 1 wt% MWCNTs) exhibits stable sensitivity, low hysteresis error, appropriate elasticity, and rapid response time, maintaining functionality over 2500 cycles. Notably, integrating PMPG sensors with a KNN algorithm enabled 100% accurate object classification by a tactile gripper, overcoming application limitations of traditional flexible sensors in complex scenarios. This work not only reveals the synergistic effect of conductive fillers and plasticizers on sensing performance but also provides a material–algorithm integrated solution for intelligent soft systems through machine learning. Future studies could focus on long-term reliability under dynamic loads and multi-modal sensing integration, promoting practical applications in wearable devices and medical robotics.

## Figures and Tables

**Table 1:** Data sheet for thermal analysis of PMPG and its components.

Sample	Pyrolysis Zone/°C	TG Peak Temperature/°C	DSC Peak Temperature/°C	Residual Rate/%
PVC	229~407 & 407~563	314	303	10.85
DBA	90~281	261	262	5.51
MWCNTs	/	/	/	/
PMPGs (1:5 1 wt%)	161~383	261	260	7.95

**Table 2:** Comparison between this work and references.

Materials	Durability	Response Time	Conductivity	Compressive Stress/Tensile Stress	Ref.
PMPG	2500 cycles	189 ms	2.5 × 10^−5^ S·cm^−1^	273 kPa/−	This work
P (AM/LMA)	2400 cycles	/	/	/	[55]
PDMS (silica@polyaniline core–shell particle)	300 cycles	300 ms	1.84 S·cm^−1^	1398 kPa/−	[56]
PAM-oxCNTs	1200 cycles	/	/	21 kPa/−	[57]
PHEMA/PPy gel	300 cycles	300 ms	67 S·cm^−1^	−/710 kPa	[58]
PB-Ag/TA@CNC	/	~70 ms	3.5 × 10^−5^ S·cm^−1^	/	[59]
PDA (GEL/AgNWs)	/	/	6 × 10^−6^ S·cm^−1^	/	[60]

## Data Availability

Data are available as asked.

## Supplementary Materials

The following supporting information can be downloaded at: https://www.mdpi.com/article/10.3390/biomimetics10060363/s1, Table S1. The compositions of PMPGs. Table S2. The EDX data of PMPG (PVC:DBA = 1:5, 1 wt% of MWCNTs). Table S3. The nonlinearity errors of PMPGs. Table S4. Specimen parameters and hysteresis errors for hysteresis characterization experiments. Table S5. Statistics of the fitting results of electrochemical impedance spectral parameters of PMPG (PVC: DBA = 1:3). Table S6. Statistics of the fitting results of electrochemical impedance spectral parameters of PMPG (PVC: DBA = 1:5). Table S7. Statistics of the fitting results of electrochemical impedance spectral parameters of PMPG (PVC: DBA = 1:7). Table S8. Statistics of the fitting results of electrochemical impedance spectral parameters of PMPG (PVC: DBA = 1:9). Table S9. Linear fitting results for conductivity of PMPGs. Table S10. Young’s modulus of PMPGs. Table S11. Toughness of PMPGs. Figure S1. The TG-DSC results of PVC, DBA, and MWCNTs. Figure S2. XRD pattern of PMPG. Figure S3. The EDX results of PMPG (PVC:DBA = 1:5, 1 wt% of MWCNTs). Figure S4. Compression characteristics of PMPGs with different MWCNTs contents. (a) PVC:DBA = 1:3, (b) PVC:DBA = 1:5, (c) PVC:DBA = 1:7, (d) PVC:DBA = 1:9. Figure S5. Curve of output voltage versus corresponding strain. PVC:DBA = 1:3, (b) PVC:DBA = 1:5, (c) PVC:DBA = 1:7, (d) PVC:DBA = 1:9. Figure S6. Curve of sensitivity versus corresponding strain. (a) PVC:DBA = 1:3, (b) PVC:DBA = 1:7, (c) PVC:DBA = 1:9. Figure S7. Electrical hysteresis characteristic curves of PMPGs. Samples (a), (b), (c), (d) differ in PVC to DBA ratio, samples (b), (e), (f), (g) differ in MWCNTs content, samples (b), (h), (i) differ in strain. Figure S8. Output characteristics at different compression speed. Figure S9. Electrochemical impedance spectra and fitting results of electrode systems with different component contents of PMPGs. (a) impedance Nyquist plot, (b) impedance Bode plot, PVC:DBA = 1:3, (c) impedance Nyquist plot, (d) impedance Bode plot, PVC:DBA = 1:5, (e) impedance Nyquist plot, (f) impedance Bode plot, PVC:DBA = 1:7, (g) impedance Nyquist plot, (h) impedance Bode plot, PVC:DBA = 1:9.
