# Supplementary material for: A Piezoelectric Sensor Based on MWCNT-Enhanced Polyvinyl Chloride Gel for Contact Perception of Grippers
Source: Biomimetics (Basel). 2025 Jun 3;10(6):363. doi: 10.3390/biomimetics10060363 (PMC12190710; doi:10.3390/biomimetics10060363)
Supplement: Supplementary file 1 [file biomimetics-10-00363-s001.zip › biomimetics-3593826-supplementary.pdf]

Table S1. The compositions of PMPGs.

	The ratio of MWCNTs			
PVC:DBA=1:3	0.5 wt%	1 wt%	1.5 wt%	2 wt%
PVC:DBA=1:5	0.5 wt%	1 wt%	1.5 wt%	2 wt%
PVC:DBA=1:7	0.5 wt%	1 wt%	1.5 wt%	2 wt%
PVC:DBA=1:9	0.5 wt%	1 wt%	1.5 wt%	2 wt%

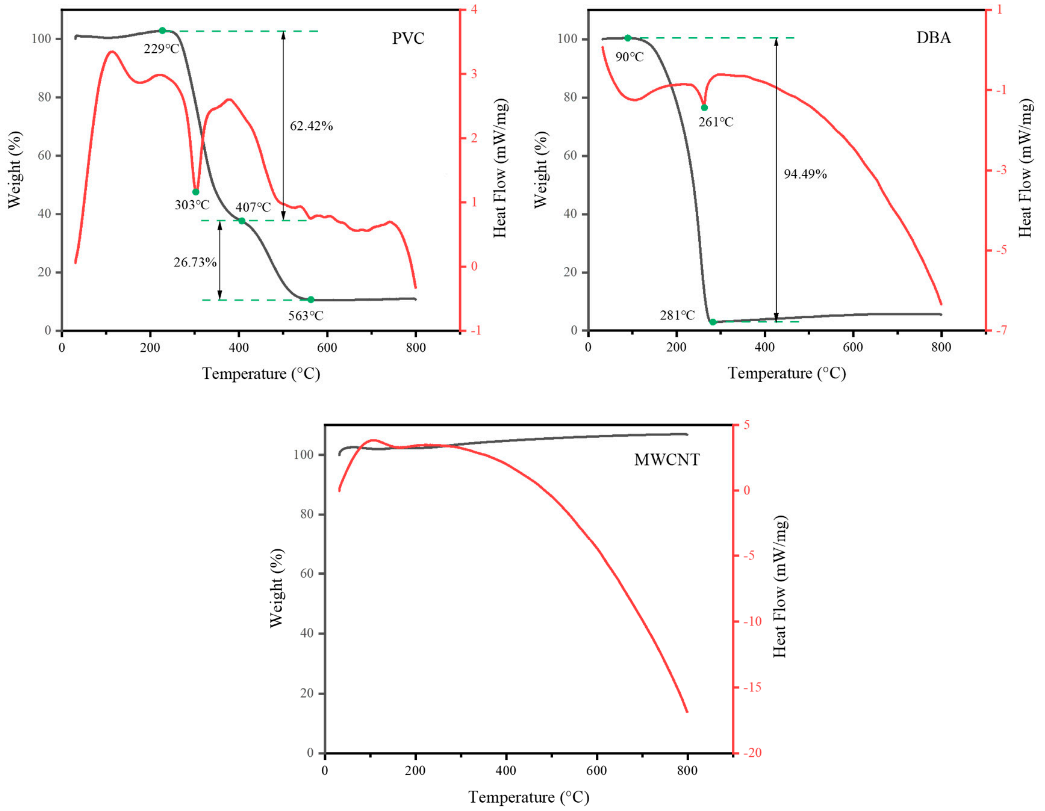

Figure S1. The TG-DSC results of PVC, DBA, and MWCNTs.

 https://doi.org/10.3390/biomimetics10060363

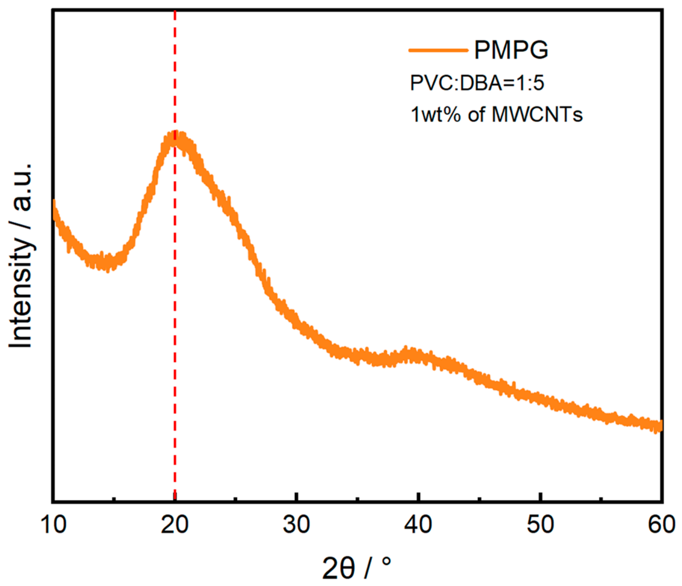

Figure S2. XRD pattern of PMPG.

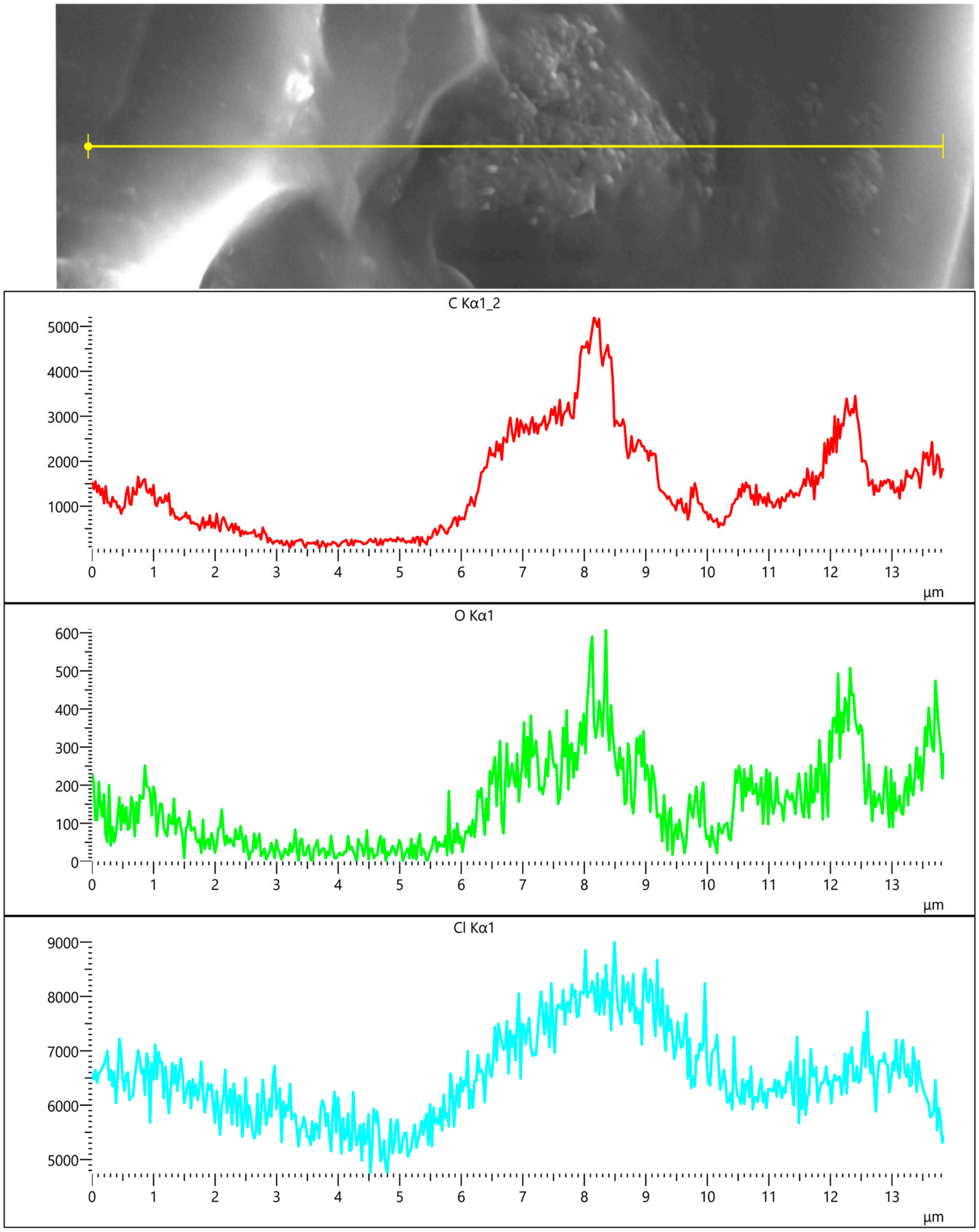

Figure S3. The EDX results of PMPG（PVC:DBA=1:5, 1 wt% of MWCNTs）.

Table S2. The EDX data of PMPG（PVC:DBA=1:5, 1 wt% of MWCNTs）.

Element	Line Type	Apparent Concentration	k Ratio	Wt%	Wt% Sigma	Standard Label	Factory Standard
C	K series	18.80	0.1880	74.21	0.18	C Vit	Yes
O	K series	2.01	0.0067	2.74	0.09	SiO2	Yes
Cl	K series	36.69	0.3206	23.05	0.16	NaCl	Yes
Total				100.00			

The output characteristics of PMPGs with different carbon tube contents were completed, and the samples used included four PVC to DBA ratios of 1:3, 1:5, 1:7, and 1:9, respectively, which were tested by a universal tensile testing machine. The compression

cycle of the test T = 4 s, the strain is constant at 60%, the output voltage signal of PMPG under four compression cycles is intercepted versus time, and the output curve is shown in Figure S3. It can be seen that the output signal tends to increase with the increase of carbon nanotube content at a certain DBA content, i.e., the increase of carbon tube content improves the electrical conductivity of PMPG and thus leads to the increase of the output voltage. When the carbon tube content is constant, the output voltage shows an increasing trend with the increase of DBA content. However, the increase in the output signal slows down with the increase in DBA content, presumably because DBA generates polarity during the pressure process that affects the conductivity.

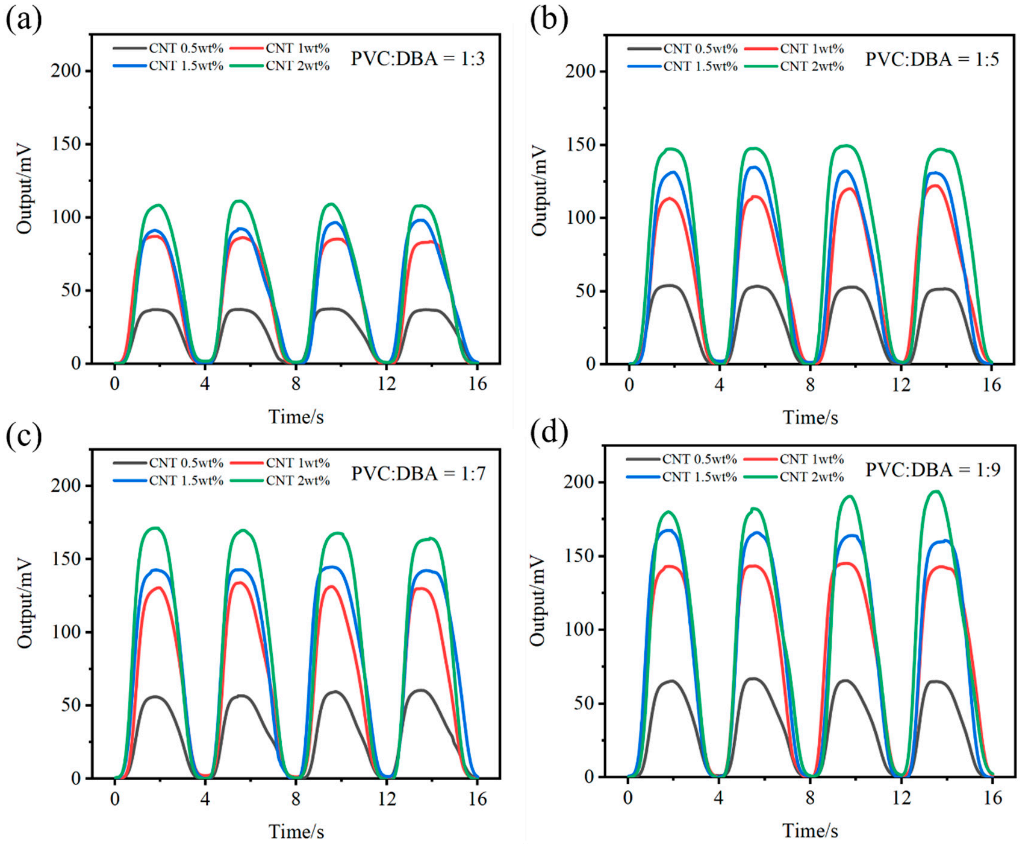

Figure S4. Compression characteristics of PMPGs with different MWCNTs contents. (a) PVC:DBA=1:3, (b) PVC:DBA=1:5, (c) PVC:DBA=1:7, (d) PVC:DBA=1:9.

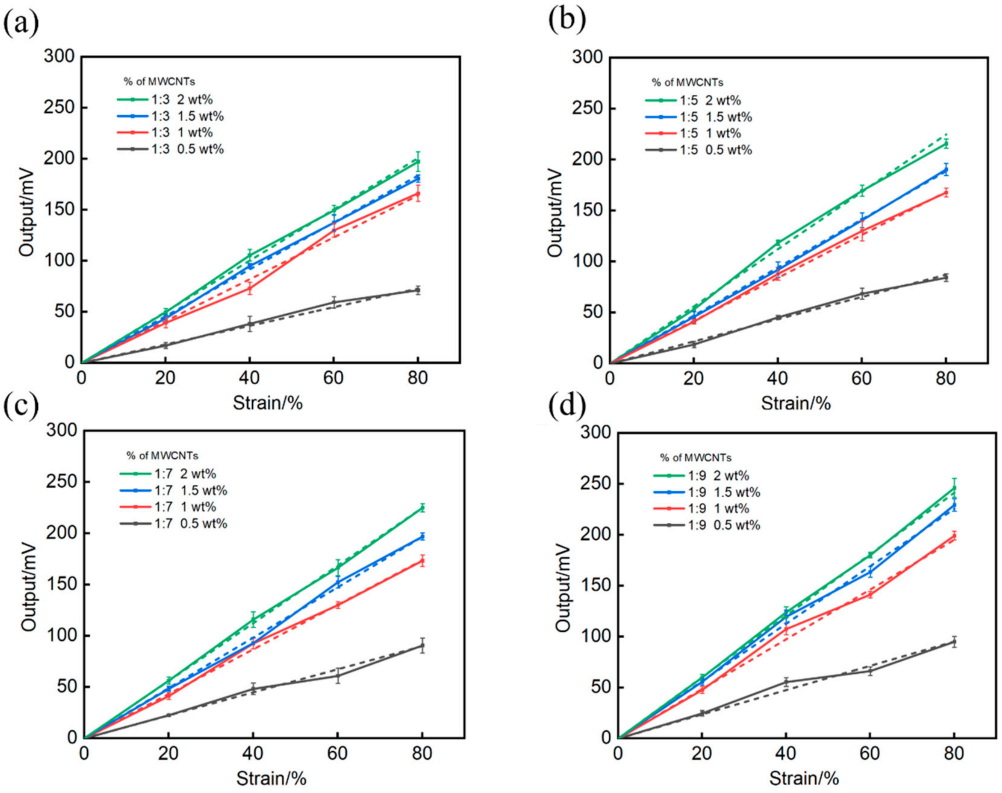

Figure S5. Curve of output voltage versus corresponding strain. PVC:DBA=1:3, (b) PVC:DBA=1:5, (c) PVC:DBA=1:7, (d) PVC:DBA=1:9.

Table S3. The nonlinearity errors of PMPGs.

	0.5 wt%	1 wt%	1.5 wt%	2 wt%
PVC:DBA=1:3	5.26%	4.73%	4.18%	3.09%
PVC:DBA=1:5	3.80%	3.32%	2.84%	1.76%
PVC:DBA=1:7	3.99%	3.05%	2.77%	2.08%
PVC:DBA=1:9	4.44%	4.21%	2.62%	1.31%

Sensitivity is the ratio between the output increment of the sensing material and the corresponding input increment in a steady state. In this paper, K is used to represent the sensitivity of PMPG. The expression of sensitivity K is as follows:

$$K = \Delta V/\varepsilon = (V-V_0)/\varepsilon \quad \text{(S1)}$$

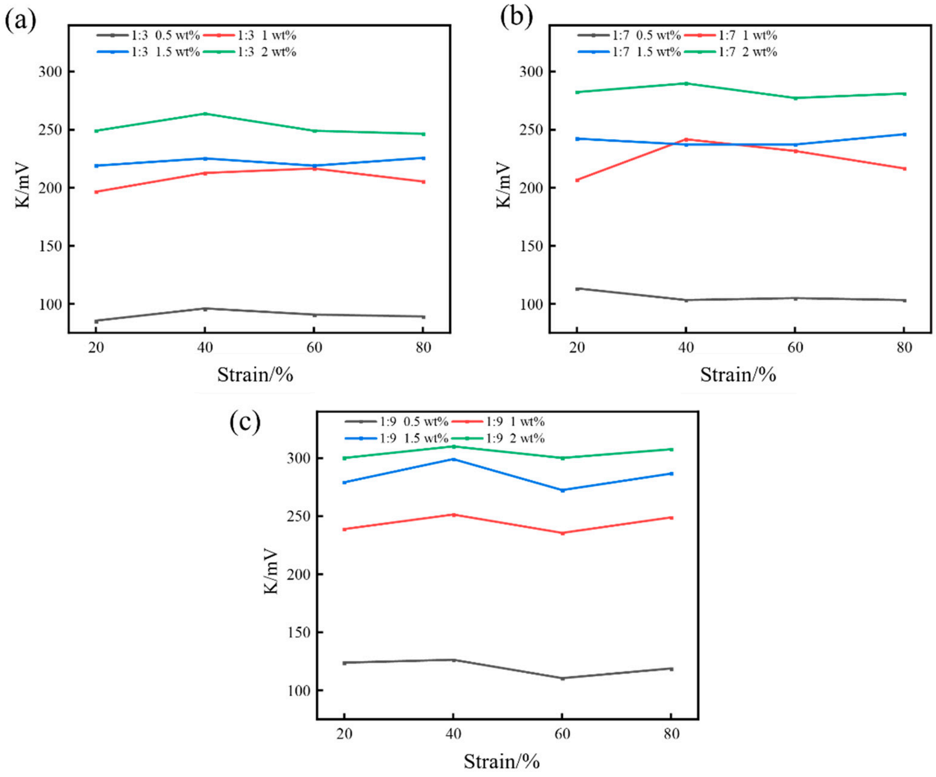

Figure S6. Curve of sensitivity versus corresponding strain. (a) PVC:DBA=1:3, (b) PVC:DBA=1:7, (c) PVC:DBA=1:9.

Hysteresis error refers to the ratio of the maximum difference between the output curves during loading and unloading to the full scale output, expressed as follows:

$$\gamma_H = \pm \Delta H_{max} / Y \times 100\%, \quad \text{(S2)}$$

where, γ_H is the hysteresis error, ΔH_{max} is the maximum difference output during loading and unloading and Y is full scale output.

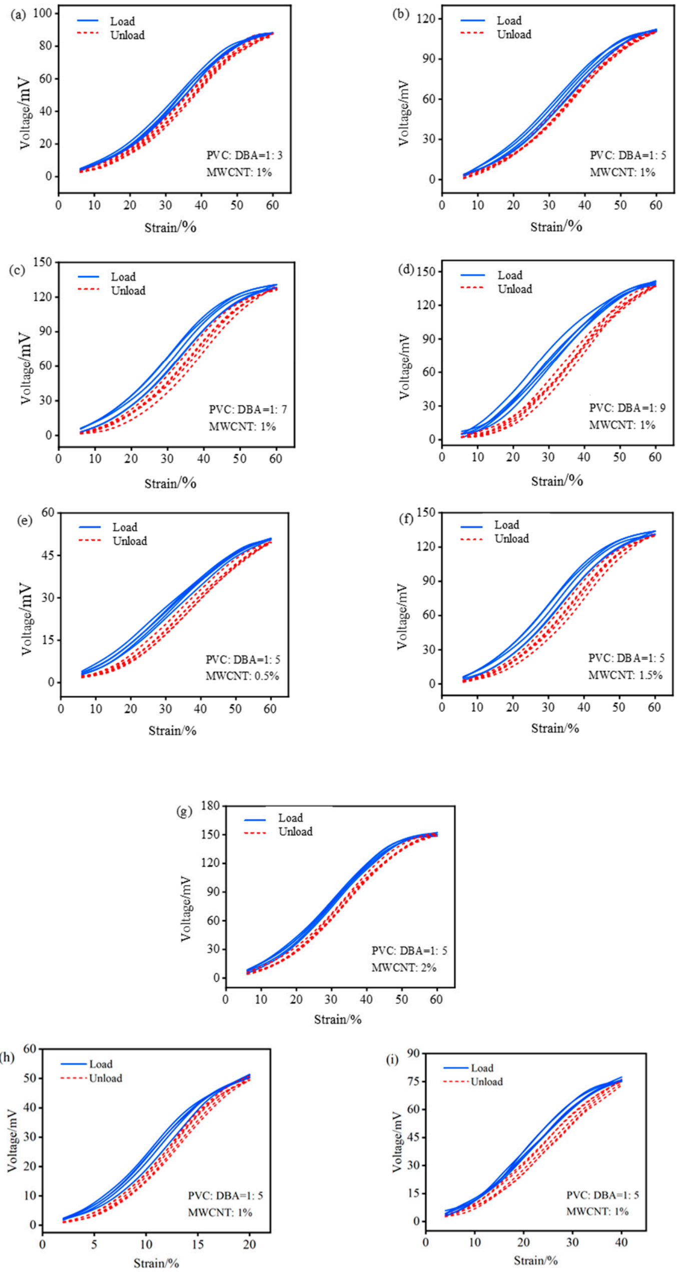

Figure S7. Electrical hysteresis characteristic curves of PMPGs. Samples (a), (b), (c), (d) differ in PVC to DBA ratio, samples (b), (e), (f), (g) differ in MWCNTs content, samples (b), (h), (i) differ in strain.

Table S4. Specimen parameters and hysteresis errors for hysteresis characterization experiments.

	**(a)**	**(b)**	**(c)**	**(d)**	**(e)**	**(f)**	**(g)**	**(h)**	**(i)**
CNT Content	1 wt%	1 wt%	1 wt%	1 wt%	0.5 wt%	1.5 wt%	2 wt%	1 wt%	1 wt%
PVC:DBA	1: 3	1: 5	1: 7	1: 9	1: 5	1: 5	1: 5	1: 5	1: 5
Strain	60%	60%	60%	60%	60%	60%	60%	20%	40%
Average hysteresis error	8.37%	6.62%	7.42%	10.19%	7.44%	6.53%	5.51%	5.64%	6.44%

To further analyze the response characteristics of PMPGs under different compression speeds, the stability of PMPGs under four compression speeds is also experimentally tested in this paper. The compression strain is set to be 60 %, and four gradient compression and recovery cycles are set to be 3 s, 2 s, 1 s, and 0.5 s, respectively. For the same compression speeds, the output signals are intercepted from four of these compression cycles versus time, and the results are shown in Figure S7. The variation of the compression speed will have a certain effect on the output signal. With the increase of the speed, there is a certain fluctuation of the output voltage signal, which may be caused by the mechanical characteristics of PMPG and the displacement error in the experiment, and the deformation generated by the sensing gel cannot be quickly recovered during the rapid loading and unloading process.

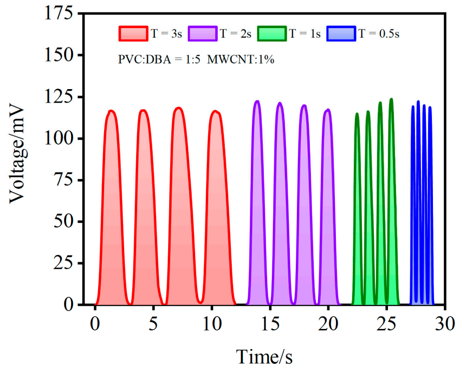

Figure S8. Output characteristics at different compression speed.

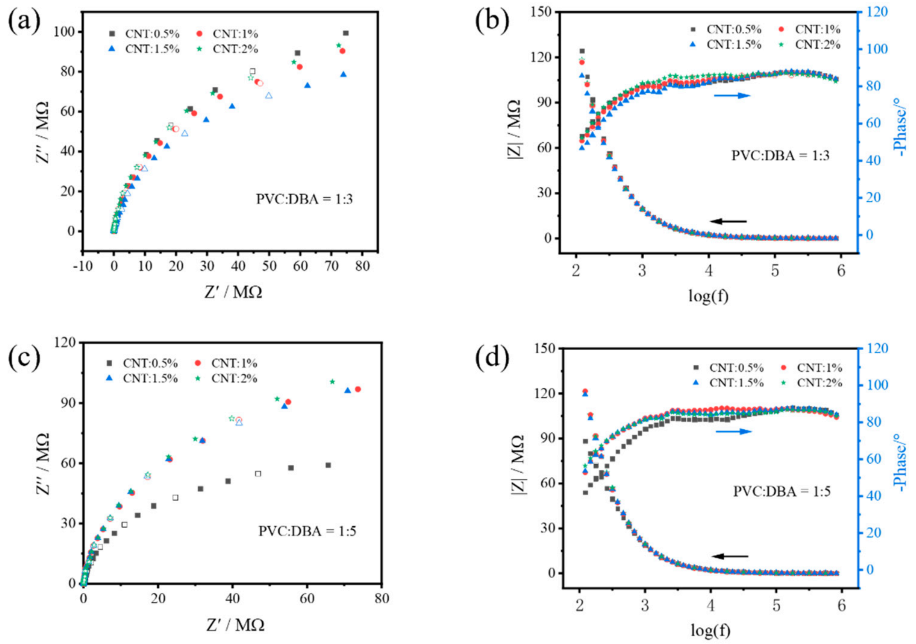

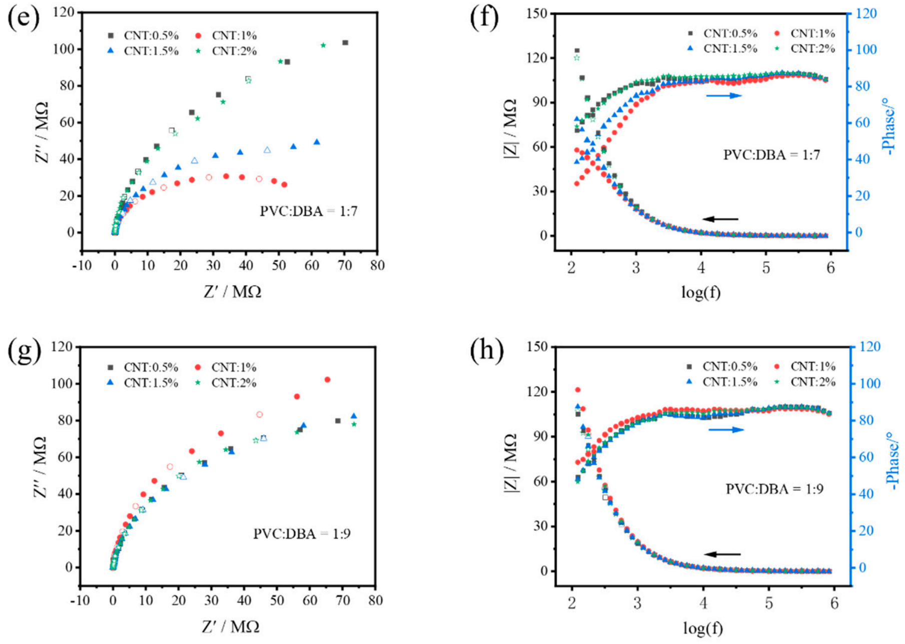

Figure S9. Electrochemical impedance spectra and fitting results of electrode systems with different component contents of PMPGs. (a) impedance Nyquist plot, (b) impedance Bode plot, PVC:DBA=1:3, (c) impedance Nyquist plot, (d) impedance Bode plot, PVC:DBA=1:5, (e) impedance Nyquist plot, (f) impedance Bode plot, PVC:DBA=1:7, (g) impedance Nyquist plot, (h) impedance Bode plot, PVC:DBA=1:9.

Table S5. Statistics of the fitting results of electrochemical impedance spectral parameters of PMPG(PVC: DBA=1:3).

	MWCNTs	CPE-P	n	Rp
PVC: DBA=1: 3	0.5%	1.24E-11	9.46E-01	2.17E+08
	1%	1.20E-11	9.49E-01	1.85E+08
	1.5%	1.26E-11	9.55E-01	1.54E+08
	2%	1.06E-11	9.72E-01	1.90E+08

Table S6. Statistics of the fitting results of electrochemical impedance spectral parameters of PMPG(PVC: DBA=1:5).

	MWCNTs	CPE-P	n	Rp
PVC: DBA=1: 5	0.5%	1.32E-11	9.48E-01	1.19E+08
	1%	9.91E-12	9.61E-01	2.05E+08
	1.5%	1.09E-11	9.68E-01	2.13E+08
	2%	1.15E-11	9.80E-01	2.33E+08

Table S7. Statistics of the fitting results of electrochemical impedance spectral parameters of PMPG(PVC: DBA=1:7).

	MWCNTs	CPE-P	n	Rp
PVC: DBA=1: 7	0.5%	1.95E-12	9.49E-01	2.50E+08
	1%	1.31E-11	9.55E-01	6.63E+07
	1.5%	1.26E-11	9.56E-01	9.48E+07
	2%	1.05E-11	9.72E-01	2.17E+08

Table S8. Statistics of the fitting results of electrochemical impedance spectral parameters of PMPG(PVC: DBA=1:9).

	MWCNTs	CPE-P	n	Rp
PVC: DBA=1: 9	0.5%	1.26E-11	9.51E-01	1.73E+08
	1%	1.03E-11	9.53E-01	2.16E+08
	1.5%	1.24E-11	9.63E-01	1.67E+08
	2%	1.16E-11	9.72E-01	1.64E+08

Here, CPE-P is the constant phase angle element, n is the index of the constant phase angle element, and Rp is the polarization resistance.

The formula for calculating the conductivity of PMPG [61]:

$$\sigma_c = \delta/(R_t \times S_c), \quad \text{(S3)}$$

where σ_c is the conductivity of PMPG, δ is the thickness of PMPG specimen, R_t is the intrinsic resistance of the sensing gel, S_c is the contact area between PMPG specimen and the electrode. Table S9 shows the calculated conductivity of PMPG, and the data fitting results are shown in Figure 3i. It can be found that the doping of carbon tubes can increase the conductivity of PMPG. At a carbon tube content of 2 wt%, the conductivity of PMPGs ranged from 10.6 μS/cm ~ 25.4 μS/cm, which was more than thirty times higher than that of PVC gels without added carbon tubes (0.31 ~ 0.36 μS/cm). When the DBA content was certain, the conductivity of PMPG increased with the increase of the carbon tube content, which confirms the previous conclusion that increasing the mass fraction of carbon tubes can increase the conductive ability of PMPG. By linear fitting of the conductivity of PMPGs, the results showed that the conductivity of PMPGs had obvious linear characteristics with MWCNTs contents at 0.5 wt%, 1 wt%, 1.5 wt% and 2 wt%, respectively. At a certain content of carbon tubes, the conductivity of PMPG increases firstly and then decreases with the increase of DBA content. And there is no obvious linear characteristic of the conductivity of PMPG with DBA content, which may be because the higher the DBA content, the lower the density of the carbon tubes in the PVC polymer network, which weakened the conductivity of the sensing material.

Table S9. Linear fitting results for conductivity of PMPGs.

	Average value of conductivity（μS/cm）			
	CNT: 0.5%	CNT: 1%	CNT: 1.5%	CNT: 2%
PVC:DBA=1:3	5.53	7.20	11.1	13.4
PVC:DBA=1:5	13.3	14.5	19.3	25.4
PVC:DBA=1:7	9.73	11.0	13.7	14.7
PVC:DBA=1:9	2.89	5.41	7.47	10.6

Table S10. Young's modulus of PMPGs.

	Average value of Young's modulus（kPa）			
	CNT: 0.5%	CNT: 1%	CNT: 1.5%	CNT: 2%
PVC:DBA=1:3	62.4	65.0	69.4	71.3
PVC:DBA=1:5	47.6	49.0	52.5	56.2
PVC:DBA=1:7	23.2	25.4	28.8	31.2
PVC:DBA=1:9	15.8	17.0	18.4	21.0

Table S11. Toughness of PMPGs.

	Average value of toughness（kJ/m^3）			
	CNT: 0.5%	CNT: 1%	CNT: 1.5%	CNT: 2%
PVC:DBA=1:3	21.7	28.8	34.1	55.3
PVC:DBA=1:5	14.9	22.6	27.4	32.4
PVC:DBA=1:7	15.6	16.9	22.6	26.7
PVC:DBA=1:9	6.7	7.8	9.9	11.0

 https://doi.org/10.3390/biomimetics10060363
